# Bringing Antimicrobial Strategies to a New Level: The Quorum Sensing System as a Target to Control *Streptococcus suis*

**DOI:** 10.3390/life12122006

**Published:** 2022-12-01

**Authors:** Bingqian Xue, Yamin Shen, Jing Zuo, Dong Song, Qingying Fan, Xiaoling Zhang, Li Yi, Yang Wang

**Affiliations:** 1 College of Animal Science and Technology, Henan University of Science and Technology, Luoyang 471000, China; 2 Key Laboratory of Molecular Pathogen and Immunology of Animal of Luoyang, Luoyang 471000, China; 3 College of Life Science, Luoyang Normal University, Luoyang 471000, China

**Keywords:** *Streptococcus suis*, quorum sensing, quorum sensing inhibitor, natural products

## Abstract

*Streptococcus suis (S. suis)* is an important zoonotic pathogen. It mainly uses quorum sensing (QS) to adapt to complex and changeable environments. QS is a universal cell-to-cell communication system that has been widely studied for its physiological functions, including the regulation of bacterial adhesion, virulence, and biofilm formation. Quorum sensing inhibitors (QSIs) are highly effective at interfering with the QS system and bacteria have trouble developing resistance to them. We review the current research status of the *S. suis* LuxS/AI-2 QS system and QSIs. Studies showed that by inhibiting the formation of AI-2, targeting the LuxS protein, inhibiting the expression of *luxs* gene can control the LuxS/AI-2 QS system of *S. suis*. Other potential QSIs targets are summarized, which may be preventing and treating *S. suis* infections, including AI-2 production, transmission, LuxS protein, blockage of AI-2 binding to receptors, AI-2-mediated QS. Since antibiotics are becoming increasingly ineffective due to the emergence of resistant bacteria, including *S. suis*, it is thus critical to find new antibacterial drugs with different mechanisms of action. QSIs provide hope for the development of such drugs.

## 1. Introduction

Quorum sensing (QS) is an intercellular communication system used by bacteria to regulate and control many important survival processes. Bacteria transfer signal molecules to each other to regulate their behavior, including virulence factor production, biofilm formation, and gene regulation [1], in a cell density-dependent manner through the QS system [2]. The QS system is divided into four types due to different signal molecules. The QS system mediated by N-Acyl homoserine lactones (AHLs) is also called the AI-1 QS system. It is regulated by the *LuxI/LuxR* gene and mainly exists in Gram negative bacteria [3]. The QS system of Gram positive bacteria is mediated by autoinducing peptides (AIP). Bacteria require specialized proteins to transport AIP inwards and outwards [4]. The QS system mediated by Autoinducer-2 (AI-2) and LuxS protein is also called the LuxS/AI-2 QS system [5]. It is the fourth kind of QS system belonging to the bacterial interspecific communication. The interspecific QS system is the AI-3 QS system, which is mediated by adrenaline and noradrenaline [5].

*Streptococcus suis* (*S. suis*) is a Gram-positive bacterium and an emerging zoonotic agent. Pig tonsils and the upper respiratory tract are major reservoir niches for *S. suis*. It can cause meningitis, septicemia, pneumonia, arthritis, or sudden death in pigs [6]. The production of virulence factors and the formation of biofilms are under the control of QS, and *S. suis* is no exception [7]. Quorum sensing inhibitors (QSIs) target QS systems; they rely on the inhibition of the communication and virulence factors rather than on killing or inhibiting the growth of the microorganisms [8]. Many studies focus on the QSIs of the LuxS/AI-2 QS system. Although there are many mechanisms that have not been thoroughly studied, the main targets have been discovered, including the inhibition of LuxS protein activity, AI-2 transmission, and *luxS* gene expression [9,10]. Compared with traditional antibiotics, interfering with QS systems is a novel means to combat *S. suis*, meaning that it is much easier for the human to resist drug resistance. This review summarizes the bacterial LuxS/AI-2 QS system and the QSI for *S. suis*. According to several ways of inhibiting AI-2, some potential strategies of inhibiting *S. suis* are proposed. It is critical to find new antibacterial drugs with different mechanisms of action.

## 2. LuxS/AI-2 QS System

**Figure 1:**
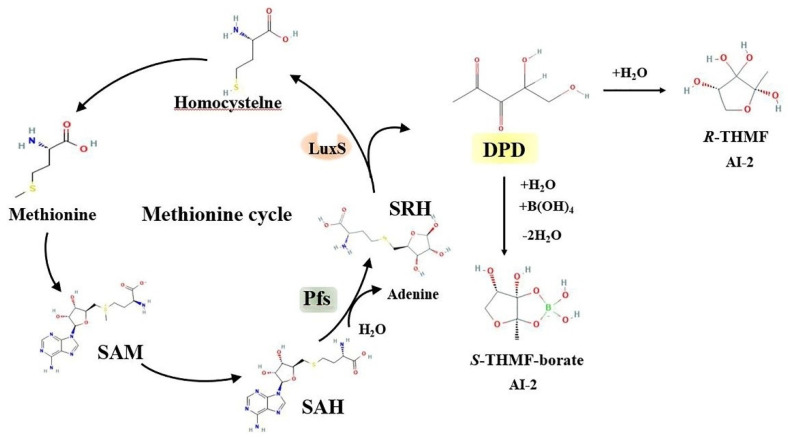
AI-2 signal molecule synthesis pathway. In bacteria, AI-2 is synthesized from S-adenosylmethionine (SAM), a chemical that is closely related to metabolism. SAM can transfer methyl groups to different substrates to generate S-adenosylhomocysteine (SAH). SAH is catalyzed by 5′ methylthioadenosine nucleosidase (Pfs) to convert it sequentially into adenine and S-ribosylhomocysteine (SRH). LuxS, as a key enzyme in the methylation cycle, can catalyze SRH to form homocysteine. In the catalytic process, a precursor molecule 4,5 dihydroxy 2,3 pentanedione (DPD) of the AI-2 molecule will be formed. DPD is decomposed into R-THMF or the S-THMF-borate AI-2 signal molecule through cyclization and rearrangement.

In a microbial community, bacteria interact with their neighbors of the same or different species. They live in symbiosis and communicate with each other using signal molecules. In general, bacteria sense chemical signaling molecules and regulate gene expression through the QS system mediated by AI-2 and LuxS, which enables bacterial communities to interact and adapt [11]. LuxS, as a key enzyme in the methylation cycle, can catalyze S-ribosylhomocysteine (SRH) to form homocysteine. In the catalytic process, a precursor molecule 4,5 dihydroxy 2,3 pentanedione (DPD) of the AI-2 molecule will be formed. DPD is decomposed into AI-2 through cyclization and rearrangement [12]. The number and behavior of bacteria are mainly mediated by an AI-2 interspecific QS system, which is widely distributed among different bacteria [13]. The importance of the LuxS/AI-2 QS system has been demonstrated by numerous studies, which showed that LuxS/AI-2 is involved in physiological processes such as biofilm formation, virulence, and antibiotic resistance [14,15]. Two receptor proteins that recognize AI-2 have been identified. One of these was found in *Vibrio harveyi* (*V. harveyi*), which regulates the behavior of the bacterium by binding a signaling molecule to the LuxP protein to detect AI-2 in the cytoplasm. The other is that AI-2 signal molecules in *Escherichia coli (E. coli)* and *Salmonella typhimurium (S. typhimurium)* recognize LsrB protein and regulate bacterial behaviors [16]. Figure 1 shows the molecule synthesis pathway for the AI-2 signal.

### 2.1. Streptococcus suis LuxS/AI-2 QS System

*S. suis* can synthesize functional AI-2 molecules while the group behavior of it is regulated by the LuxS/AI-2 QS system. This QS system can affect the growth, metabolism, biofilm formation, drug resistance, and virulence of *S. suis* [17]. Our research group has extensively studied the LuxS/AI-2 QS system of *S. suis* and found that all strains harbor the *luxS* gene and secrete and produce AI-2 signal molecules [18]. We cloned and expressed the LuxS protein, analyzed its three-dimensional fine structure (PDB index number 4XCH), and found that Zn^2+^ is the main component of the active center of the LuxS protein of *S. suis* [19]. Site-directed mutagenesis showed that two amino acid mutations have a major impact on substrate binding and enzyme catalytic ability. They also affect the production of AI-2 in *S. suis* and the formation of a biofilm. A lack of the *luxS* gene has many effects on the hemolytic activity, biofilm formation, adhesion, toxic force, toxic force genes, and inflammatory factors of *S. suis* [20]. The *luxS* gene deletion strain of *S. suis* lost the ability to produce AI-2 signal molecules, while the overexpression strain did not increase AI-2 molecule levels. We also showed that LuxS/AI-2 has an effect on bacterial resistance to quinolones by affecting the SatAB efflux pump, upregulating the expression of *tet (M)*, increasing the resistance of *S. suis* to tetracycline, and affecting bacterial resistance by regulating the formation of a biofilm [21].

### 2.2. LuxS/AI-2 QS System Participates in the Regulation of Bacterial Function

Although the LuxS/AI-2 QS system is widely present in both gram-positive and gram-negative bacteria, the biological functions involved in regulation vary slightly among different bacteria. For example, *V. harveyi* mainly regulates bioluminescence and colony morphology. It is involved in the production and release of virulence factors in *V. cholerae* [22,23]. It is involved in biofilm formation and the expression of virulence factors in *E. coli* [24].

For most bacteria, the regulatory function of the LuxS/AI-2 QS system is mainly reflected in three aspects, including bacterial virulence, biofilm, and other functions [25]. Zhang et al. constructed the *luxS* deletion mutant and its complemented strain from *Haemophilus parasuis (H. parasuis) serovar 2*, and preliminarily investigated the effects of *luxS* gene on several virulence-associated properties. The comparison of ΔluxS with *H. parasuis* 2 and C-luxS strains revealed that the *luxS* gene was related to growth characteristic, stress response, QS, biofilm formation, adherence, and virulence-associated LD_50_ and tissue burdens of bacteria [26]. Sun constructed a *luxS* gene mutant strain to identify how LuxS/AI-2 affects pathogenicity. The results showed that the AI-2 activities of *Edwardsiella piscicida* (*E. piscicida*) were 56-fold higher than those in the ΔluxS strain, suggesting that the *luxS* gene promotes AI-2 production. Another outcome demonstrated that the deletion of the *luxS* gene in EIB202 reduced its ability for growth, mobility, biofilm formation and for the infection of zebrafish. Results indicate that the LuxS/AI-2 QS system in *E. piscicida* promotes its pathogenicity through an increase in a diverse array of physiological activities [27]. Lu found that *luxS* gene deletion could lead to the decreased virulence of *Streptococus mitis* biofilm, reduced biofilm synthesis, and a loose structure. The level of drug resistance of the *luxS* deletion strain to ampicillin, ciprofloxacin and tetracycline decreased. These results indicated that the LuxS/AI-2 QS system could not only affect the formation of *Streptococcus* biofilm, but also affect its drug resistance [28]. These studies show that the LuxS/AI-2 QS system is biologically important as it is involved in numerous physiological processes. Many researchers disrupt the cellular communication between bacteria at the QS system level.

## 3. Quorum Sensing Inhibitors in *Streptococcus suis*

QSIs are promising alternatives to antibiotics for treating bacterial infections. QSIs of *S. suis* are being studied for their ability to attenuate bacterial virulence, biofilm formation, and because bacteria may have more trouble developing resistance to them.

Many previous studies showed that by inhibiting the formation of AI-2, targeting the LuxS protein, inhibiting the expression of the *luxS* gene can control the LuxS/AI-2 QS system of *S. suis.* The synthesis of AI-2 in *S. suis* can be used as a research target to search for and develop many QSIs. Current research focuses on the inhibition of *luxS* gene expression and the inhibition of the AI-2 metabolic cycle. QSIs have a strong inhibitory effect on bacterial growth and some can be extracted from whole plants or parts of flowers, fruits, and roots. Emodin (1,2,8-trihydroxy-6-methylanthraquinone) is a natural compound extracted from the roots of rhubarb, which has anti-inflammatory, antibacterial, anti-biofilm formation [10,29], and other properties. *luxS* is a key gene in the LuxS/AI-2 QS system. Emodin can decrease the levels of AI-2 signal molecules and reduce the formation of *S. suis* biofilms mainly by down-regulating *luxS* gene expression, thus inhibiting the catalytic activity of LuxS [10]. Antimicrobial peptides (AMPs), are secreted by the immune system and participate in the first line of defense. They have great potential to combat microbial infections by directly killing or inhibiting bacterial activity and/or by modulating the immune response of the host [30]. In the early stage of the study, our research group synthesized several antimicrobial peptides, including the *S. suis* QS system blocking peptide TTMHSIRTNRHN, which can inhibit AI-2 synthesis in *S. suis* in vitro [31]. The inhibitory polypeptide HSIRTGSKKPVPIIY, which has a significant antibacterial effect, is a new type of broad-spectrum antibacterial polypeptide that can inhibit the production of AI-2 by *S. suis*, *Staphylococcus aureus (S. aureus)*, *E. coli*, and other bacteria [32]. Treating *S. suis* with the synthetic antimicrobial peptides temprine-La (S)(T-La(S)), temprine-La (Fs) (T-La (Fs)), RGD-T-La(S), or RGD-T-La (Fs) down-regulates the *FBPS*, *luxS*, and *GAPDH* genes and significantly reduces the concentration of AI-2 [33]. Han developed a novel *S. suis* QSI that targets the LuxS protein using phage display technology. A peptide inhibitor (TNRHNPHHLHHV) was used to interact with the LuxS protein following three rounds of phage display panning [9]. This approach generates high affinity peptide inhibitors that can inhibit enzyme activity. High affinity peptide inhibitors provide a new avenue for the development of novel antibacterial drugs. Our research group discovered a natural product, paeoniflorin, which has the same active site as the *luxS* gene in *S. suis* and forms protein-ligand interactions with the key amino acid residues of the LuxS protein, significantly reducing AI-2 production [34]. In the present review, we summarize QSIs of *S. suis* (Table 1).

## 4. Potential Drug Target of LuxS/AI-2 QS System to Inhibit *Streptococcus suis*

### 4.1. Potential Drug Target: AI-2 Production

AI-2 controls virulence factors and virulence production in host models for various pathogens, including *E. coli*, *Helicobacter pylori*, *Streptococcus pneumoniae* and *Vibrio cholerae (V. cholerae)*. This suggests that virulence inhibitors targeting AI-2 signaling may have a relatively wide range.

In the process of the biosynthesis of autoinducers, 5′-Methylthioadenosine/S-adenosylhomo-cysteine nucleosidase (MTAN) generally performs a crucial role in maintaining homeostasis in bacteria [36]. MTAN catalyzes the depurination of 5’-methylthioadenosine (MTA) and S-adenosylhomocysteine (SAH) to generate 5′-methylthioribose (MTR) or S-ribosylhomocysteine (SRH), respectively. The inhibition of MTAN blocks the generation of SRH and reduces the generation of AI-2 signal molecules. The transformation of MTA is also blocked, resulting in the accumulation of MTA. PhT-ImmA is a series of QSIs that target MTAN enzymes and block the production of SAH in *Streptococcus pneumoniae* [36]. This decreases the rate of synthesis of AI-2. In addition, they found MT-DADMe-Immucillin-A, EtT-DADMe-Immucillin-A and BuT-DADMe-Immucillin-A, which are inhibitors of *V. cholerae* MTAN (VcMTAN). MT- and BuT-DADMe-Immucillin-A inhibited AI-2 production in *E. coli O157:H7*. BuT-DADMe-Immucillin-A inhibition of AI-2 production in both strains persisted for several generations and resulted in the reduction in biofilm formation. Consequently, MTAN inhibitors can suppress the biological synthesis of AI-2, thereby disrupting QS [37]. It has now become the main strategy for the development of new drugs for treating *S. suis* infections. Various AI-2 homologs can also significantly inhibit MTAN activity, interfere with AI-2 synthesis, and block the QS system [38]. For instance, 3-(Dibromomethyl) isobenzofuran-1 (3H)-ketone derivatives, which have a similar ring structure to AI-2, also have a significant inhibitory effect on AI-2 activity [39]. Furthermore, rutin [40], citral [41], furanocoumarin [42], and other plant extracts can also reduce the synthesis of AI-2, thus reducing biofilm formation. Decreasing the virulence of bacteria by inhibiting AI-2 synthesis reduces the likelihood that bacteria will develop drug resistance.

### 4.2. Potential Drug Target: AI-2 Transmission

The degradation of AI-2 interrupts bacterial communication and regulates bacterial invasiveness. The inhibition of its activity by natural products or chemical substances can block the transfer of AI-2 molecules. It has been reported that andrographolide, the main component of Andrographis, a genus of flowering plants in the family acanthaceae, can significantly reduce the AI-2 activity of *E. coli* in poultry [43], inhibit the transcription levels of QS regulatory genes, and reduce the expression of virulence genes. 2(5H)-Furanone can disrupt the QS system of *Campylobacter jejuni* and reduce the activity of AI-2 [44]. Chemicals can also interfere with AI-2 delivery. For example, AI-2 production by *V. harveyi* is reduced by 70% after treatments with high concentrations of α-isophthalic acid and by 10% after a treatment with N-benzylcinnamamide. Both of these chemicals can modulate physiological functions by interfering with bacterial AI-2 communication [45]. The AI-2 kinase LsrK in *E. coli* can phosphorylate AI-2 in vitro and, if LsrK-treated AI-2 is added to *E. coli*, the regulatory capacity of the QS system is significantly reduced [46]. To date, no natural or synthetic substances have been found that degrade AI-2 in *S. suis*. However, naturally stimulated enzymatic quenching of the QS system, which is a novel way to control bacterial physiological functions, could lead to the development of synthetic QSIs to quench the QS system of *S. suis*.

### 4.3. Potential Drug Target: LuxS Protein

LuxS is a mandatory enzyme in the biosynthesis of DPD and encloses in Gram-positive and Gram-negative bacteria. A class of SRH analogues has been shown to inhibit *Bacillus subtilis* proliferation by targeting the LuxS protein [47]. S-anhydroribosyl-L-homocysteine and S-homoribosyl-L-cysteine are SRH analogs with a significant inhibitory effect on LuxS protein activity [48]. The high-affinity inhibitor may open up new approaches to the development of therapeutic drugs. Meng experimented with LuxS inhibitors from natural products for *Lactobacillus reuteri*. The results showed that norathyriol, mangiferin, baicalein, and kaempferol had good binding ability to LuxS protein. It has been shown that mangiferin may be a potential inhibitor of the LuxS protein [49].

### 4.4. Potential Drug Target: Blockage of AI-2 Binding to Receptors

AI-2 signaling molecule analogues prevent signaling molecules from binding to receptors and have been developed in many laboratories around the world. Most are artificially modified competitive inhibitors of bacterial QS signal molecules based on the structure of natural signal molecules as templates [50]. Phenothiazines and other compounds may prevent receptors from recognizing signal molecules by interfering with the binding of AI-2 to the receptors [51]. Some substances can bind to receptors, occupying sites and acting as false signaling molecules. For example, N,N’-bisalkylimidazolium salt can compete with AI-2 for receptors, which greatly reduces the recognition rate [52]. In *Vibrio species*, LuxP is the AI-2 binding protein. Wang discovered a series of aromatic diols; they would form a boric acid complex and mimic the structural features of DPD-boric acid complex in binding with LuxP. In addition, 1-alkylated DPD analogs compete with phosphorylated AI-2 and directly target the QS system, preventing AI-2 from binding to the LsrR repressor, leading to the expression of the *lsr* repressor operon [53]. Although the AI-2 receptor in *S. suis* has not been identified, this suppression strategy opens up new avenues for the development of novel drugs.

### 4.5. Potential Drug Target: AI-2-Mediated QS

**Figure 2:**
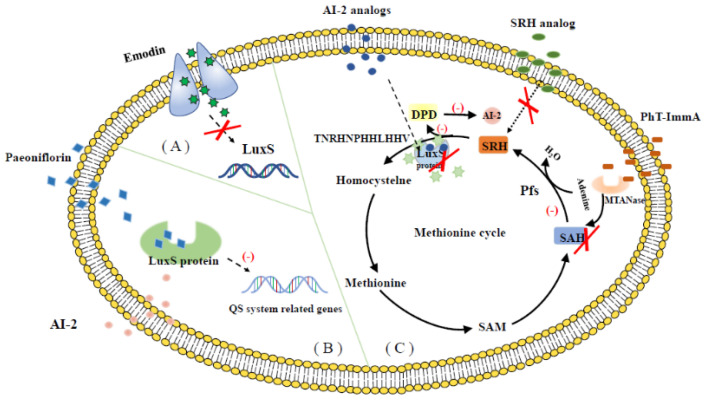
The action mechanisms of QSIs. (**A**) Emodin can decrease the levels of AI-2 signal molecules by down-regulating *luxS* gene expression, thus changing the phenotypes of the bacteria. (**B**) Paeoniflorin competes with AI-2 for LuxS protein, and paeoniflorin can interact with key amino acid residues of LuxS protein forming protein-ligand to inhibit AI-2 binding to LuxS. (**C**) AI-2 is a metabolite of the SAM cycle, and its synthesis depends on LuxS and Pfs. During AI-2 synthesis, SAM forms SAH under the catalysis of methyl transferase, and SAH degrades into SRH and adenine under the action of Pfs. Subsequently, SRH is catalyzed by LuxS to generate DPD and homocysteine. DPD is the precursor molecule of AI-2 and has high activity, which can cyclize itself to form AI-2. The resulting Homocysteine is catalyzed by MetF/K to get methyl regenerated and SAM again enters the metabolic cycle. PhT-ImmA targets MTAN enzymes and blocks the production of SAH, and the synthesis rate of AI-2 was significantly reduced. The SRH analog, as a pseudosignal molecule, cannot participate in the catalytic synthesis of DPD by LuxS, resulting in a decrease in the synthesis rate of AI-2. AI-2 analogs and TNRHNPHHLHHV directly acted on LuxS protein, which decreased the activity of LuxS protein, inhibited the synthesis of DPD, and reduced the synthesis of AI-2.

The search for and development of safe, non-toxic, active QSIs is a rapidly growing field of research. Many natural products found in plants, fruits, and vegetables also act on the QS systems of pathogens, including naringin [54] and coumarin [42]. Although natural QSIs have been reported in many studies, their inhibition rates are generally low. However, synthetic QSIs can overcome this limitation. A variety of synthetic aromatic sulfones can directly inhibit the QS system of *V. harveyi* [55]. Linoleic acid, palmitic acid, and other substances can also act on the QS system, inhibiting bacterial interspecific regulation and significantly preventing biofilm formation. Cinnamon essential oil can interfere with the AI-2-mediated QS system, increasing its susceptibility to antibiotics [56]. Apigenin and hexadecanoic acid can directly inhibit the QS system mediated by AI-2 signaling molecules [57]. DPD is a key compound in the AI-2-mediated QS pathway. Janda and co-workers have synthesized a series of DPD analogs, which are not only active against the QS of *V.harveyi*, but also are active against the AI-2 QS of *S. typhimurium* [58]. Another study reported that fimbrolide's natural product (5Z)-4-bromo-5-(bromomethylene)-3-butyl-2(5H)-furanone has been considered to be a potent antagonist of both the AHL and AI-2 system in several organisms [26,59,60]. In the present review, we summarize potential QSIs of *S. suis* (Table 2 and Table 3). See Figure 2 for the action mechanisms of QSIs.

## 5. Conclusions and Future Perspectives

QS is defined as a cell-to-cell communication process that bacteria use to coordinate group behaviors [70]. The ability of QS to regulate cell-to-cell communication plays an important role in bacterial virulence and drug resistance. Researchers have focused a great deal of attention on the QS system with the aim of reducing bacterial virulence and developing new anti-infective therapies. *S. suis* is a pathogen that is a major cause of economic losses in the pig industry. This is why the goal of finding new treatments for bacterial infections has focused the attention of researchers on targeting QS and developing QSIs. There is growing evidence that QSIs act on QS systems through different pathways and that QSIs can efficiently regulate the physiological functions of bacteria. The QSI of *S. suis* is being studied. At present, the main ways are to inhibit the *luxS* gene, block the formation of AI-2, and interfere with the binding of signal molecules with the LuxS protein. This result will reduce the virulence of bacteria, biofilm formation, and it may be more difficult for bacteria to develop drug resistance. The intense interest in this field of research has led to the discovery of other types of QSIs, including bacteriocins and probiotic secretions [71]. There are, however, also some issues worth investigating in the study and application of QSIs. First, in many studies that describe the QSI activity of natural and synthetic compounds, most of this research is based on experiments with quorum sensing signal molecule reporter strains. However, there are uncertainties regarding some QSIs. The adverse effects of certain naturally occurring active substances on the host cannot be excluded. Thus, researchers should perform adequate control experiments and should carefully assess the toxicity of the compounds in the bacterial species they are working on, and their reliability in clinical applications must be confirmed [72,73]. However, the trial period is long and it is not practical to apply it to clinical use earlier. Second, QSIs affect bacteria not by inhibiting their growth, but by regulating their gene expression or bacterial behavior. In most studies, it is used as an antibacterial synergist to dissolve naturally extracted or artificially synthesized QSIs in solvent and act on bacteria [10]. However, there is no unified answer to how to use QSIs, which we explore next. Although some QSIs exhibit the problems described above, their ability to regulate bacterial functions cannot be ignored. More in-depth research on the QS system and QSIs are required to better prevent and treat bacterial diseases such as *S. suis* infections. It is believed that more natural, non-toxic and effective QSIs will be developed and put into production in the near future to make it an effective choice for controlling human, animal and plant pathogen contamination. It will also benefit agriculture, fisheries and other industries susceptible to microbial pollution, playing an irreplaceable role.

## Figures and Tables

**Table 1:** *S. suis* quorum sensing inhibitors.

QSI	Source	Mechanism	References
Emodin	Rhubarb	Down-regulates the *luxS* gene, inhibits AI-2 synthesis	[10]
TTMHSIRTNRHN	Synthetic	Inhibits AI-2 synthesis	[31,32]
HSIRTGSKKPVPIIY			
TNRHNPHHLHHV			
Temprine-La(s)(T-La(s))	Synthetic	Decreases AI-2 concentrations and down-regulates biofilm-related genes	[33]
Temprine-La(Fs)(T-La(Fs))			
RGD-T-La(s)			
RGD-T-La(Fs)			
Paeoniflorin	Peony	Reduces the binding rate of AI-2 to receptors	[34]
Coptis water extract	Huanglian	Inhibits the expression of the *luxS*, *gdh*, *fbps*, and *mrp* genes	[35]

**Table 2:** Potential natural products quorum sensing inhibitors of *S. suis*.

QSI	Source	Bacteria	Mechanism	References
Andrographolide	Andrographis	*Escherichia coli*	Decreases AI-2 activity	[38]
Citral	Lemon	*Vibrio harveyi*	Reduces the synthesis of AI-2, inhibits the formation of biofilm	[41]
Furanocoumarin	Grapefruit	*Vibrio harveyi*	Reduces the synthesis of AI-2	[42]
mangiferin	Mango	*Lactobacillus reuteri*	inhibitor of LuxS protein	[49]
Naringin	Grapefruit	*Vibrio harveyi*	Inhibits the AI-2-mediated QS system	[54]
Cassia	Cassia oil	*Escherichia coli*	Inhibits the production of AI-2	[56]
Hexadecanoic acid	Black pepper	*Vibrio harveyi*	Suppresses the QS system	[57]
Apigenin	Celery	*Vibrio harveyi*	Inhibits the AI-2-mediated QS system	[61]
Fructose furoate	Plants	*Escherichia coli*	Inhibits the expression of QS system-related genes (*fimA*, *csgA*, *espA*)	[62]
D-Galactose	Lactose	*Vibrio harveyi*	Reduces the synthesis of AI-2	[63]
Ajoene	Garlic	*Pseudomonas aeruginosa*	Suppresses the QS system	[64]

**Table 3:** Potential synthetic quorum sensing inhibitors of *S. suis*.

QSI	Structure Diagram	Bacteria	Mechanism	References
p-TolT-ImmA	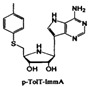	*Streptococcus* *pneumoniae*	Inhibits the MTAN enzyme, blocks the production of SRH and AI-2 signal molecules	[36,65]
EtT-ImmA	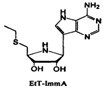			
MeT-ImmA	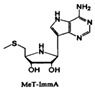	*Streptococcus* *pneumoniae*	Inhibits the MTAN enzyme, blocks the production of SRH and AI-2 signal molecules	[36,65]
p-C1-PhT-DADMe-ImmA	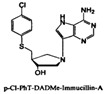	*Vibrio cholerae* *Escherichia coli O157:H7* *Streptococcus pneumoniae*		
MT-DADMe-immucillin-A	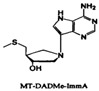			
EtT-DADMe-immucillin-A	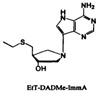	*Vibrio cholerae*		
BuT-DADMe-immucillin-A	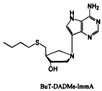	*Vibrio cholerae* *Escherichia coli* *O157:H7*		
S-anhydroribosyl-L-homocysteine	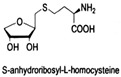	unknown	SRH analogues, as substrates of the LuxS protein, exhibit inhibitory activity against the LuxS protein	[48,66]
S-homoribosyl-L-cysteine	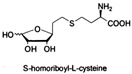			
Aromatic sulfones	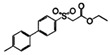	*Vibrio harveyi*	Suppresses the AI-2 QS system	[55]
Sulfones(S-1)	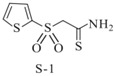			
Sulfones(S-2)	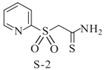			
SRH analog-1	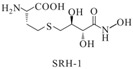	*Bacillus subtilis*	Reversible competitive inhibitors of *luxS*	
SRH analog-2	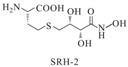	*Bacillus subtilis*		[47]
SRH analogs	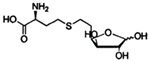	*Vibrio harveyi*		[67]
2-Methylpropyl-DPD	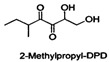	*Vibrio harveyi* *Salmonella typhimurium*	Inhibits the AI-2-mediated QS system	[53]
Isopropyl-DPD	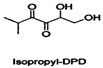			
Neopentyl-DPD	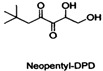	*Vibrio harveyi* *Salmonella typhimurium*	Inhibits the AI-2-mediated QS system	[53]
Pentyl-DPD	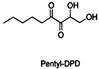			
Linoleic acid	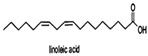	*Vibrio harveyi*	Inhibits AI-2 QS and the formation of bacterial biofilms	[68]
Oleic acid	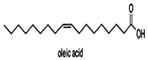			
Palmitic acid	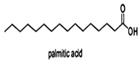			
3, 4-dibromo-2 (5H)-furanone	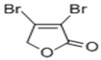	*Campylobacter jejuni*	Interfere with QS system activity	[69]
2(5H)-furanone	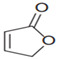		Inhibiting the activity of AI-2	[44]

## Data Availability

The data that support the findings of this study are available from the authors upon reasonable request.
